# Dysfunctional neural dynamics associated with sensory phenotypes in Fragile X syndrome: insights from mouse models

**DOI:** 10.1186/s11689-025-09634-4

**Published:** 2025-12-30

**Authors:** Anubhuti Goel, Khaleel A. Razak, Alexander A. Chubykin, Michelle W. Antoine

**Affiliations:** 1 Department of Psychology, University of California, Riverside, Riverside, CA 92521 USA; 2 Graduate Neuroscience Program, University of California, Riverside, CA 92521 USA; 3 Department of Biological Sciences, Purdue Institute for Integrative Neuroscience, Purdue Autism Research Center, Purdue University, West Lafayette, IN 47907 USA; 4 Section on Neural Circuits, National Institute of Alcohol Abuse and Alcoholism, National Institutes of Health, Bethesda, MD 20892 USA

## Abstract

Fragile X Syndrome (FXS), the leading known inherited cause of atypical behaviors associated with autism spectrum disorders (ASD), arises due to the reduced expression or absence of the Fragile X Messenger Ribonucleoprotein 1 (FMRP). Individuals with ASD and FXS often experience atypical sensory processing across modalities such as touch, hearing, and/or vision. The consequences of altered sensory processing can be debilitating, leading to impairments in sensory discrimination and an inability to filter out irrelevant sensory stimuli such as innocuous sounds, smells, sights, or touches. Currently, there is a significant knowledge gap in the field of FXS regarding the circuit mechanisms that drive atypical sensory processing and how these contribute to hypersensitivity and secondary effects, such as learning impairments and increased anxiety. Animal models of FXS mirror many of the sensory hypersensitivity issues observed in humans, exhibiting heightened anxiety, as well as learning and social impairments. Here, we discuss the dysfunctional neural dynamics underlying atypical sensory processing across modalities in FXS, potential therapeutic interventions targeting specific ion channels, receptors, and circuits, and propose future research directions that could pave the way for circuit-targeted therapies.

The brain continuously processes sensory input to guide behavior, and anomalies in sensory experiences can lead to atypical perception, learning, memory and cognition. Sensory abnormalities, such as over- or under- reactivity to stimuli or an unusual interest in sensory aspects of the environment, are common diagnostic criteria for ASD, with up to 90% of patients exhibiting abnormal sensory behaviors/experiences [1–8].

Various expressions are used to describe sensory anomalies in ASD. Before discussing sensory dysfunction and associated neural deficits, we first provide an overview of the terms commonly used to describe behavioral and emotional responses to sensory stimulation in ASD:

- Hypersensitivity: Over-responsiveness, more reactive, or an exaggerated behavioral response to sensory stimulation compared to typically developing children (TDC). Examples include:
  1. Overreaction to loud sounds or repetitive irrelevant sounds (e.g., air conditioner noise)
  2. Overreaction to flashing lights
  3. Overreaction to sensations like cold, heat, pain, tickling, and avoidance of going barefoot, especially in sand or grass
- Hyposensitivity (Under-responsiveness): Lack of or reduced behavioral response to sensory stimulation compared to TDC. Also described as sensory-seeking or low-registration, where individuals crave or seek intense sensory stimulation or experiences. Examples include:
  1. Tuning out or suppressing sounds and visual inputs, suppressing targets in the visually attended area by fixating on the center of a visual scene rather than individual objects.
  2. Seeming oblivious of wet or dirty diapers, messy face or hands, and diminished response to cold, heat, pain, tickle and itch.
  3. Frequently touching people and objects, fidgeting, or repeating noises
- Impaired habituation: The failure to habituate to sensory inputs. Examples include sensory overload and difficulty processing multiple sensory inputs simultaneously.
- Tactile defensiveness: Negative reactions such as rubbing, scratching, negative expressions, withdrawal, or avoidance in response to tactile stimulation [9]. Tactile defensiveness is associated with enhanced response and slower habituation rates to a repeated tactile stimuli [10, 11]. Examples include:
  1. Avoiding hugs or pats on the back or resisting being held.
  2. Rubbing or scratching areas touched or withdrawal from splashing water.
  3. Distress during grooming activities like haircuts, face washing, fingernail cutting, or hair washing.

Although sensory processing abnormalities are common to ASD, affecting ~ 69% to 90% of preschoolers with ASD [12], they manifest in diverse ways. About 39% of patients exhibit hypo-responsiveness, 19% hyper-responsiveness, and 36% display mixed responsiveness to sensory stimuli [13]. These sensory abnormalities can be debilitating, contributing to anxiety, social deficits, attention difficulties, and delayed/impaired learning [14–18]. For example, if a person with ASD perceives normal stimuli as overwhelming or cannot filter out irrelevant stimuli, they may limit social interactions (avoiding eye contact or hugging), control sensory inputs through rituals, and experience delays in learning and adapting to environmental changes.

Methylation and transcriptional silencing of the *Fragile X Messenger Ribonucleoprotein 1* (*Fmr1)* gene, due to the abnormal expansion of the trinucleotide CGG repeats in the promoter to over 200, leads to loss of encoded protein (FMRP), resulting in Fragile X syndrome (FXS) [19]. FXS is one of the most common single-gene causes of ASD with intellectual disability (ID) [19–21]. Approximately 7–30% of individuals with FXS have ASD [22].

FMRP is an mRNA-binding protein that negatively regulates the translation of many mRNAs important for neuronal development and synaptic function in response to neuronal activity [23, 24]. Despite identifying the genetic cause of FXS, our understanding of how abnormal communication results in the diverse symptoms of ASD and FXS remains incomplete. The large degree of heterogeneity in neuropathological and genetic features in ASD suggests that the fundamental deficit may not be any specific cellular pathology but rather a perturbation in network properties emerging from neuronal interactions [25].

Mouse models have been instrumental in exploring cellular and circuit-level defects. One well established model is the *Fragile X* Messenger Ribonucleoprotein 1 *gene* (*Fmr1)* knockout (KO) mouse. The mouse *Fmr1* gene product shares 97% homology with human FMRP, including conservation of the CGG repeats [26]. Animal models like mice allow for granular recording methods and a broader and more nuanced toolkit of experimental manipulations. Additionally, *Fmr1* KO mice exhibit functional and behavioral alterations similar to those seen in humans, including sensory abnormalities and some ASD-related perseverative phenotypes [27, 28]. For a detailed review of additional mouse models of FXS and their pre-clinical relevance see [29].

Atypical sensory experiences in individuals with FXS and ASD span multiple sensory modalities, including taste [7], touch, audition, smell and vision [30]. In this review, we focus on the more frequently reported sensory issues across three modalities–visual, auditory, and somatosensory, in both mice and humans, delineating common threads in the associated neural dysfunction prevalent across audition, vision and touch. Although altered sensitivity to taste and smell has been observed in FXS individuals and investigated in *Fmr1* KO mice [31–33], these modalities fall outside the focus of this present review. Given the emphasis on dysfunctional inhibition as a key underlying cause of many FXS symptoms (and likely ASD), we describe potential therapeutic interventions targeting specific ion channels, receptors, and circuits, and offer insights for future research directions.

## Auditory modality

### Similarities in structure and mechanisms for basic auditory processing in humans and mice

Early auditory processing involves similar pathways and mechanisms across mammals, including rodents and humans [34–37]. Rodents, including mice, depend on auditory function for nocturnal predator detection, sound localization and social communication, including ultrasonic vocalizations. The gross organization of the auditory pathway from the cochlea to the primary auditory cortex is conserved. This has led to mice being a useful model system to study a wide range of human auditory disorders including hearing loss and central auditory processing deficits [38, 39]. The basilar membrane of the cochlea is organized tonotopically in humans and mice with lower frequencies represented close to the apex of the basilar membrane [40]. The main difference is the range of audible frequencies extends into ultrasonic range in mice compared to humans. The monaural and binaural pathways through the brainstem and midbrain are largely similar between mice and humans leading to generalized principles of location and spectrotemporal encoding across species [41, 42]. Likewise, the descending pathways within the auditory system are also mostly similar in structure and function across species [43]. The different divisions of the auditory thalamus, including the multisensory medial division, the lemniscal ventral and the non-lemniscal dorsal divisions are similarly present in mice and primates [44]. The primary auditory cortex is organized tonotopically in mice and primates, including humans [45–47]. There are multiple additional cortical regions, which is where the divergence in complexity of auditory processing may emerge across species [48]. However, most of the research in FXS has focused on low level auditory processing, including responses to tones, noise and spectrotemporal modulations leading to identification of remarkably similar phenotypes across species as described below.

### Auditory hypersensitivity in FXS linked to altered neural rhythms in cortex

Genetic abnormalities are associated with abnormal language development across a number of neurodevelopmental disorders. One of the most debilitating and consistent phenotypes in children with FXS is auditory hypersensitivity. Auditory hypersensitivity, either alone or in combination with other FXS psychopathologies such as hyperarousal and hyperactivity, may lead to high anxiety and abnormal language development in FXS [49]. Thus, understanding the circuit mechanisms underlying abnormal auditory sensitivity is crucial. It is also important to develop translationally relevant biomarkers in the auditory domain to facilitate the pre-clinical to clinical therapeutic development pipeline.

In the past 8–10 years, several studies have focused on auditory processing in humans with FXS and in rodent models of FXS, including mice and rats. Similar to humans with FXS, *Fmr1 KO* mice also exhibit severe behavioral auditory hypersensitivity, which manifests behaviorally as a strong propensity for audiogenic seizures and abnormal acoustic startle responses. Given this shared sensitivity to sounds, recent studies have explored potential underlying mechanisms using electrophysiological recordings. Overall, these investigations have revealed remarkably similar cortical hyperexcitability and auditory temporal processing deficits that may underlie auditory hypersensitivity and language deficits in FXS.

#### 1. Elevated N1 peak amplitude in EEG recordings from human and animal models of FXS suggests a hyper-responsive auditory system.

Electroencephalograph (EEG) recordings in patients with FXS and typically developing controls have revealed several consistent phenotypes. The earliest studies focused on auditory event-related potentials (aERP). Which are derived from EEGs or magnetoencephalographs (MEGs) by extracting signals time-locked to auditory stimuli. aERP consists of distinct peaks, the first three of which are termed P1, N1 and P2. These peaks occur at specific latencies and reflect processing in largely distinct brain circuits, including thalamocortical inputs (P1), cortical processing (N1), and arousal-related brain regions (P2). aERP studies have consistently reported enhanced peak amplitudes in humans with FXS [50–56], particularly in the first negative peak (N1) amplitude. This suggests abnormally elevated cortical responses and/or increased synchrony of neuronal activity. Supporting this, neuroimaging studies show that the superior temporal gyrus —a brain region crucial for processing speech, language, and sound recognition— exhibits reduced volume but enhanced activation in FXS [57–60].

Magnetoencephalograph (MEG) recordings, with better spatial resolution than EEG, also show consistently elevated first negative peak amplitude [61]. Similarities in findings using EEG and MEG, across a number of studies with heterogenous ages and FXS characteristics, increase the confidence that there is a fundamental difference in sensitivity to basic sounds in FXS. Not only is aERP peak amplitude increased, but there is also reduced habituation of peak amplitudes to repeated sounds in FXS [54, 62]. The temporal lobe in FXS also shows white matter enlargement, which may contribute to abnormal responses [63]. Taken together, the aERP studies in FXS indicate a hyper-responsive auditory system which may result from underlying temporal lobe anatomical abnormalities.

Remarkably similar EEG phenotypes have been observed in the *Fmr1* KO mouse. Lovelace et al. [64] reported elevated aERP amplitudes, and Lovelace et al. [65] demonstrated reduced aERP amplitude habituation in the auditory cortex of anesthetized mice. [66] recorded from developing *Fmr1* KO mice at postnatal days (P) 21 and 30, showing that these phenotypes emerge early in development.

#### 2. Increased gamma band power may contribute to a mixed phenotype of hyperarousal, auditory hypersensitivity, and social impairments.

While the earlier work on aERP in FXS focused on ‘time x amplitude’ domain response analyses, EEG waveforms can be analyzed in the spectral domains to generate a sophisticated quantification of underlying neural oscillations that are thought to be involved in sensory and cognitive functions [67, 68]. Both baseline EEG and sound-evoked responses can be analyzed in the spectral domain. When comparing baseline resting power in different EEG frequency bands between individuals with FXS and typically developing controls, a consistent finding is elevated gamma band (30–100 Hz) power. This was first shown in young FXS subjects (mean age ~ 26 years) [69], where elevated gamma power was correlated with social and sensory difficulties, as assessed using the Social Communication Questionnaire (SCQ) and sensory profile tests. Single-trial gamma band power (STP) was also elevated during auditory stimulation. STP measures non-phase-locked background noise during acoustic stimulation. These findings were replicated in subsequent studies [52], which showed that elevated gamma resting-state power correlates with reduced spectrotemporal processing for dynamic auditory stimuli in FXS.

Lovelace et al. [64] recorded EEGs from the auditory and frontal cortex of awake and freely moving adult mice using epidural screw electrodes. They found that awake and freely moving adult *Fmr1 KO* mice exhibited elevated broadband gamma power in resting EEGs under both still (not moving) and movement conditions compared to wild-type (WT) mice. Gamma power was even higher, when the KO mice were moving, suggesting a potential link to hyperactivity behaviors reported in these mice. This implies that the elevated gamma band power in humans may also be linked to hyperarousal and hyperactivity, which, when coupled with auditory hypersensitivity, could manifest as social or sensory impairments.

#### 3. Decreased spectrotemporal response consistency and its contribution to atypical speech and language processing

As mentioned, auditory hypersensitivity, either alone or in combination with other FXS psychopathologies such as hyperarousal and hyperactivity, may contribute to abnormal language development in FXS [49]. Language-related deficits in FXS include delays and early plateauing of language development [70], repetitive and perseverative speech, semantic errors [71], and reduced auditory short-term memory [72]. Compared to expressive language, relatively less is known about speech perception in FXS [73]. Correcting abnormal auditory sensitivity early in development may lead to long-lasting improvements in anxiety, language, and cognitive function in children with FXS.

The ability of the auditory system to process fast changes in spectral and temporal attributes of sounds is critical for speech recognition [74–77]. To probe spectrotemporal processing in FXS, studies have used a type of stimulus called the ‘chirp’. The chirp is either a tone or broadband noise (~ 2 s duration) which is 100% amplitude modulated (signal fluctuates between fully on and fully off), with the frequency of modulation increasing from 1 to 100 Hz or decreasing from 100 to 1 Hz. The chirp facilitates a rapid measurement of transient oscillatory response (delta to gamma frequency range) to auditory stimuli of varying frequencies and can be used to compare oscillatory responses in different groups in clinical and pre-clinical settings [78]. One commonly used measure of chirp response is called the intertrial phase coherence (ITPC), which measures the consistency of phase locking across trials. If the phase angles of the response relative to the stimulus is variable from trial to trial, the ITPC is low (0 is minimum value). If the phase angle is the same across trials, then the maximum ITPC of 1 is seen. Typically, in human EEG work, ITPC varies between 0.05–0.5. Humans with FXS (mean age ~ 26 years) showed reduced ITPC to the chirp signal compared to typically developing controls [79]. The reduced ITPC was in the low gamma band range (30–50 Hz). These findings have been replicated with a different cohort of patients (mean age ~ 25, [52]) and demonstrate reduced spectrotemporal response consistency in individuals with FXS. Moreover, these results suggest a possible mechanism for abnormal speech and language function. Highlighting the conservation of physiological phenotypes across humans and mice, Lovelace et al. [64] reported reduced ITPC in response to chirp stimulation in the *Fmr1* KO mouse.

#### 4. Absence of cortical lateralization and atypical speech and language processing

Croom et al. [80] showed using a gap-in-noise stimulus paradigm that the KO mice have difficulty in consistently responding to short gaps in ongoing stimuli from P21. Importantly, when tested with the 40 or 80 Hz auditory steady-state responses (ASSR) or the chirp, the left cortical hemisphere shows greater ITPC values than the right, particularly in the temporal regions where auditory cortex is located [81]. This was observed for the higher frequency temporal modulations (high gamma band in chirp and 80 Hz ASSR). The cortical asymmetries were observed in both P21 and adult mice, suggesting an early development. This is consistent with other studies of auditory cortex that reveal functional asymmetries in WT mice [82, 83]. Such cortical asymmetries are absent in the *Fmr1 KO mice*. This finding is relevant for processing of complex vocalizations, including speech in humans, as evidence exists for left hemispheric specialization for speech elements with fast temporal modulations, while the right hemisphere may be specialized for slower modulations and spectral information [84, 85]. The ‘asymmetric sampling of speech’ theory proposed that the left hemisphere is specialized for processing phonetic cues, while the right hemisphere may facilitate representation of intonation-type cues. Therefore, the absence of cortical lateralization in FXS may hinder parsing of speech signal temporal cues, and delay language development [86]. The absence of cortical lateralization in the *Fmr1 KO* mice may arise due to abnormal long distance connectivity patterns [87]. Given that temporal processing is critical for speech and language function [75], the EEG observations using temporal processing may form a useful bridge to understand the structural and circuit basis of speech impairments in ASD.

EEG data from the *Fmr1 KO* rodent and FXS humans shows similar phenotypes. Such robust, repeatable, and scalable electrophysiological responses can serve as translation relevant biomarkers to develop pre-clinical drug development and in human clinical trials. These responses may serve as outcome measures and/or stratification strategies. However, an important caveat is that the clinical relevance of such measures remains mostly understudied and should be a focus of future studies.

#### 5. Abnormal function of parvalbumin-positive neurons

The EEG data from humans with FXS and the *Fmr1 KO* mice identify consistent phenotypes in both resting gamma band power, and the phase locking consistency to gamma frequency modulations in acoustic stimuli. In addition, single trial power in the gamma band is also elevated, suggesting abnormalities in cortical circuits that generate dynamic oscillations in the 30–100 Hz range. It is important to distinguish mechanisms of low-frequency (30–60 Hz) gamma oscillations and broadband gamma power [88–90]. A decrease in low-gamma oscillations and an increase in broadband gamma power can both arise from abnormal function of parvalbumin positive (PV^+^) GABA neurons in the cortex [88, 91–93]. Additionally targeting of Kv3.1 channels, which tend to be associated with PV^+^ neurons, during a specific developmental window, improves circuit and behavioral function. Deletion of *Fmr1* in PV^+^, but not somatostatin-positive (SST^+^) neurons, resulted in abnormal anxiety and social behaviors, and dysregulated de novo protein synthesis [94].

In the auditory cortex, there is delayed development of PV^+^ GABA neuron density in *Fmr1 KO* mice [95]. There is also abnormal development of the perineuronal nets (PNN) that are associated with PV^+^ neurons in the auditory cortex. This is associated with enhanced spiking of auditory cortex individual neurons to sounds. Loss of *Fmr1* leads to elevated activity of matrix metalloprotease-9 (MMP-9). Genetic or pharmacological reduction of MMP-9 in *Fmr1 KO* mice restores PNN density on PV^+^ neurons and normalizes spiking activity of single neurons and EEG phenotypes [95–97]. Taken together, auditory sensory cortical data in *Fmr1 KO* mice show abnormal expression and function of PV^+^ GABA neurons across development. These deficits strengthen an emerging idea in the FXS field that PV dysfunction may underlie the EEG circuit dynamics seen in *Fmr1 KO* mice and FXS humans. Indeed, in support of this, targeted restoration of PV^+^ neuron function shows improved functional and behavioral outcomes.

#### 6. Reduced *Fmr1* expression in cortical and midbrain regions drives altered neural rhythms and spectrotemporal processing deficits.

The literature on sensory processing in FXS specifically, and ASDs more broadly is ‘cortex-centric’ with most studies focusing on the neocortex and hippocampus. While in vitro slice recordings provide some resolution in terms of origins of electrophysiological deficits, in vivo recordings from the cortex cannot be too sure about the origins of cortical deficits. For example, all of the cortical EEG phenotypes recorded in humans with FXS and the *Fmr1 KO* mice may originate from local circuit dysfunction and/or be inherited from subcortical sites. It remains unclear whether both the power-type deficits (resting gamma, ERP, STP) and temporal processing deficits (chirp, ASSR, gap-in-noise) arise at the same levels of the auditory system. Overall, relatively less is known about how sub-cortical sites contribute to abnormal circuit dynamics in FXS and autism. Relevant to FXS, *Fmr1* protein is expressed across multiple levels of auditory system from the brainstem to the cortex. A few studies have focused on subcortical deficits in the *Fmr1 KO* mouse (reviewed in [98]), but very little is known in humans with FXS. One approach that is fruitful in understanding cortical vs sub-cortical deficits in FXS is the use of transgenic mouse lines with spatially conditioned *FMRP* removal.

The conditional *Fmr1* mouse (Fmr1 CKO) line was developed in the laboratory of Dr. Ben Oostra [99]. In this model, the promoter and first exon of *Fmr1* are flanked by bacteriophage P1-derived loxP sites, allowing for the creation of a null allele (resulting in complete absence of FMRP) in specific cell types and at specific time points. This is achieved by crossing *Fmr1 CKO* mice with tissue-specific or inducible Cre-recombinase-expressing lines. Germline recombination of the *Fmr1 CKO* allele recapitulates several hallmark features observed in FXS patients and in the original *Fmr1* KO mouse [100], including macroorchidism [99].

The Nex-Cre and CamKII-Cre lines have been used to selectively delete *Fmr1* in forebrain excitatory neurons, while preserving FMRP expression in the thalamus, midbrain, and brainstem [101, 102]. In forebrain-specific deletion studies, the increased resting gamma power, increased STP and elevated ERP amplitudes were present in cortical EEG recordings. Hyperactivity was also seen behaviorally in the open field and elevated plus maze. However, the ITPC deficit with chirp stimuli was absent, suggesting that the reductions in spectrotemporal response consistency may originate from *Fmr1* reduction in non-cortical areas.

A complementary approach to studying the systems-level contributions of FMRP to elevated neural power and temporal processing deficits in *Fmr1* knockout mice is to examine the causal effects of *Fmr1* re-expression. The FMRP conditional restoration mouse line (referred to as *Fmr1*^loxP−Neo/y^ or Fmrp-cON) expresses only 5–15% of wild-type FMRP levels, but Cre-mediated deletion of the inserted *Neo* cassette restores FMRP expression to normal levels [103]. Holley et al. [104] used the Nse-Cre line to remove or selectively restore FMRP in the midbrain—particularly in the inferior and superior colliculi and upper brainstem—while leaving FMRP expression intact in the thalamus and forebrain. In these mice, midbrain-specific re-expression of FMRP did not rescue elevated power but did restore ITPC to chirp stimuli. Preliminary findings further suggest that midbrain-specific deletion of FMRP does not produce the hyperactivity phenotype, implicating region-specific roles of FMRP in distinct behavioral and neural phenotypes.

Together, these studies suggest a double dissociation: power anomalies and hyperactivity stem from cortical dysfunction, while temporal processing deficits arise from midbrain dysfunction following FMRP deletion. These results have major implications for pre-clinical drug development and clinical trials because the choice of outcome measures will depend on where in the circuit (forebrain vs. midbrain) a potential drug may primarily act on. Future studies with other Cre models are necessary to confirm these trends.

## Visual modality

### Similarities in the visual pathway and function in humans and mice

While the proportion of the cerebral cortex that is dedicated to processing visual information is smaller in mice, the overall structure and function of the visual cortex is conserved across mammals–specifically the hierarchical organization of the visual system [105]. Information from the retina travels to primary visual cortex in mice (striate cortex in primates and humans) and several higher order visual areas in mice (extrastriate cortex in primates and humans). Work in primates has established two parallel pathways that process visual information: the ventral and the dorsal [106, 107]. While the ventral stream is required for object recognition, the dorsal stream plays an important role in motion perception. Recent work shows that functionally the HVAs in mice serve the same roles as the extra striate cortex. Anatomical [108, 109] and functional [110] studies in mice show two subnetworks or subpopulations of HVAs that are analogous to ventral and dorsal streams in primates.

Mice devote multiple cortical areas to visual processing and rely heavily on visual information during navigation. Given the structural and functional similarities between mouse and human visual systems, mouse models of FXS have provided a valuable platform for studying visual impairments.

### Visual abnormalities in FXS

**Fig. 1:**
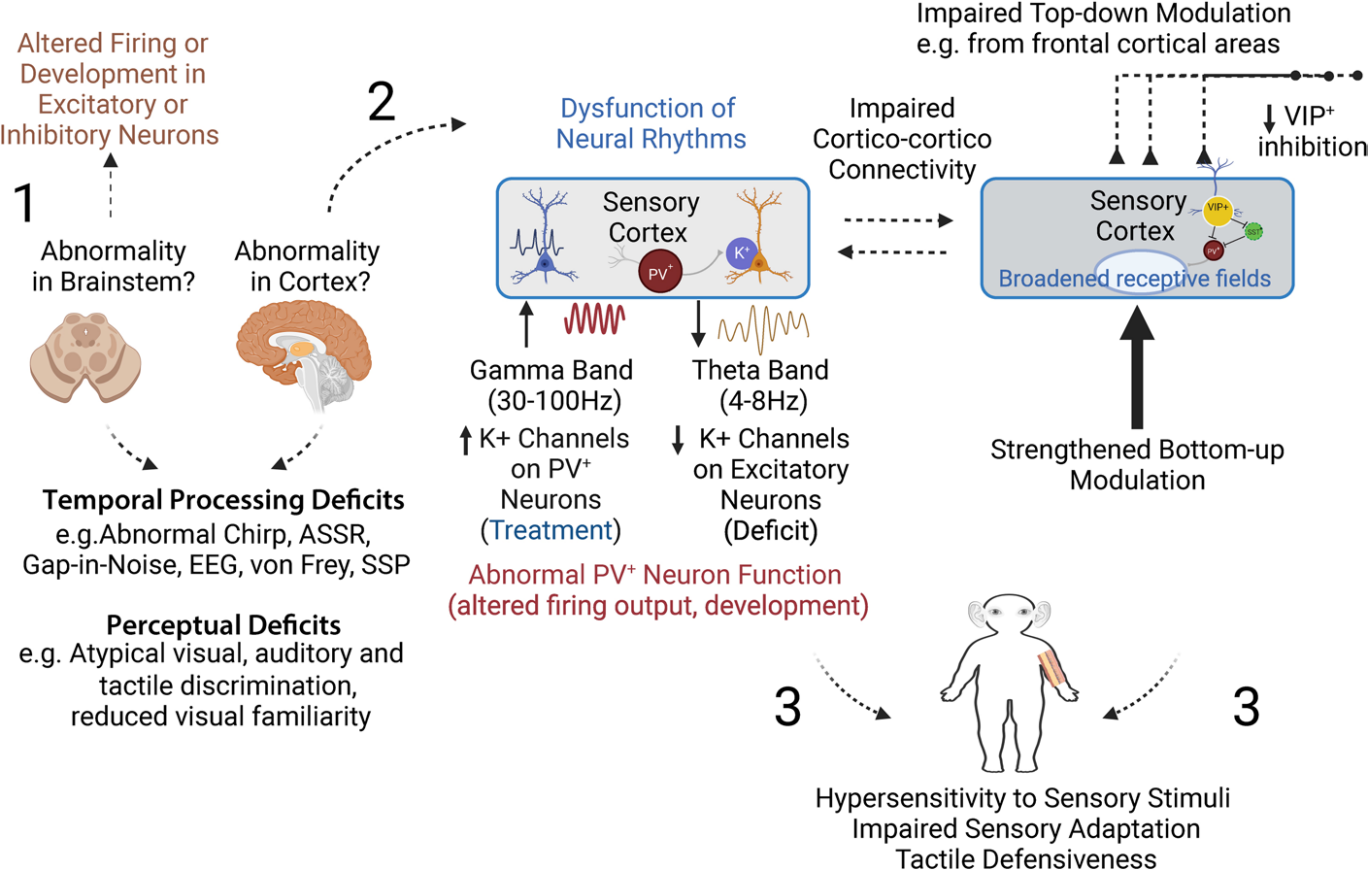
1) Several tests are used to uncover processing deficits in FXS patients, which are predicted to underlie the perceptual deficits observed. However, the ASDs literature is heavily ‘cortex-centric,’ but some basic research studies in mice suggest that primary deficits could originate in other brain areas, such as the brainstem (midbrain, pons, and medulla oblongata). 2) Summary of shared abnormalities across the auditory, visual, and somatosensory cortices that may underlie the atypical behavioral and emotional reactions in FXS patients (3)

Visual perception challenges in FXS and ASDs involve difficulties in processing and responding to visual stimuli. These impairments can significantly impair the ability of ASD patients to interact with their environment, learn new information, and engage socially. Multiple studies have reported deficits in visual and temporal perception in FXS patients [111–114]. For example, individuals with ASD often display atypical visual processing, characterized by an extensive focus on specific visual details and difficulties in integrating visual information [30, 115]. Another common deficit is the stronger image center bias irrespective of object distribution. Here individuals with ASD were shown images of different scenes, for example, a desk or a bedroom. Using gaze tracking researchers found that, compared to neurotypical controls, individuals with ASD fixated on the center of the image, irrespective of the components in the visual scene [116] (Fig. 1).

Visual impairments in FXS and ASD may also be represented by the difficulties in using visual cues to guide behavior and learning. Indeed, in FXS, there are specific challenges in visual-spatial tasks and visual discrimination learning, which are crucial for daily functioning [114]. One specific idea that has emerged is that difficulties in discrimination and learning might result from hypersensitivity to sensory stimuli. A recent study showed that, indeed, hypersensitivity to auditory stimuli prevent visual discrimination and this phenotype was captured in both humans with FXS and *Fmr1 KO* mice [117].

Individuals with ASDs similarly display challenges in visual learning, often struggling with tasks that require the perception of complex visual scenes or extraction of information from a visually cluttered environment [118]. Other studies of children with autism showed a deficit in the ability to detect coherent motion [119] and contrast detection [112]. Children with ASD also showed reduced “spatial suppression”, which is an enhancement in perceiving motion of high contrast stimuli [120]. In addition to learning, visual atypicalities can also affect motor functions. For example, visual-motor dysfunction in ASD includes disruption in drawing and constructing abstract designs [72, 121].

### Mechanisms underlying visual atypicalities in FXS

Theoretically, the neural basis for these learning impairments likely involve inefficient visual information encoding and storage in neurons. Studies using EEG in individuals with FXS have found an enhancement in the N1 component of visual stimuli, suggesting hyperexcitability in the visual processing areas of the brain [122]. This indicates neural dysfunction associated with FXS, particularly in the way the brain processes visual input. To delineate a more granular identification and examination of neural deficits related to hyperexcitability and other mechanisms at multiple scales, mouse models have been invaluable. The goal for the field is to use these mechanistic insights to drive circuit based therapies for visual abnormalities in FXS. In addition, the information gained from these studies is also applicable to ASD more broadly. In the section below we discuss insights from animal models, particularly the *Fmr1 KO* mouse, in elucidating the cellular and molecular mechanisms underlying visual dysfunctions in FXS.

#### 1. Impaired synaptic plasticity and circuit connectivity in visual areas of FXS mice

Visual perceptual impairments may result from altered connectivity and synaptic plasticity in the neural circuits involved in sensory processing, particularly in the visual cortex and associated regions. Neuroimaging studies have shown that disruptions in these areas can lead to atypical neural responses to visual stimuli [123]. Neurophysiological research has also identified synaptic anomalies in brain regions responsible for visual memory and learning, contributing to difficulties in forming and retaining visual association memories [124]. Additionally, studies in the hippocampus have demonstrated cell type-specific synaptic plasticity triggered by the oscillations [125]. Theta burst stimulation (TBS), a classical synaptic plasticity protocol to induce long-term potentiation (LTP), is triggered by theta oscillation [126]. Similarly, visually evoked theta oscillations may provide a mechanism for the induction of synaptic plasticity of the neuronal connections between the synchronized areas. Thus, if theta oscillations are necessary for synchronization between different areas and triggering synaptic plasticity of these connections, weaker and frequency-shifted oscillations may lead to a decreased effectiveness of induced synaptic plasticity. There are extensive reports of impaired short-term and long-term synaptic plasticity in *Fmr1 KO* mice [127–129]. FMRP is a transcriptional regulator upstream of many synaptic plasticity-related proteins [130, 131]. The absence of *FMRP* has been linked to changes in synaptic plasticity, including an increase in metabotropic glutamate receptor (mGluR) dependent long-term depression (LTD) in the hippocampus and the cortex and a higher threshold for LTP [132, 133]. The short-term plasticity (STP), which is critical for information processing [134] and working memory [135], was also impaired in excitatory hippocampal synapses of *Fmr1 KO* mice [136].

#### 2. Dysfunction of neural rhythms contribute to deficits in visual perception and learning

Different oscillation frequencies have been suggested to represent diverse inter-areal information flow, with higher-frequency oscillations such as gamma associated with feed-forward information, while lower-frequency oscillations such as alpha and beta associated with feedback [137–140]. Interestingly, theta oscillations represent a unique oscillation believed to represent an underlying mechanism of working memory in the prefrontal cortex. Synchronization of theta rhythms between the hippocampus and prefrontal cortex is important for the integration and processing of stored information during working memory tasks [141, 142].

Studies in mouse models suggest that memory of visual features and environmental familiarity is encoded by persistent theta (4–8 Hz) frequency oscillations in local field potentials and neuronal activity within the visual areas of the brain. These oscillations, particularly within the theta frequency range, play a crucial role in processing and maintaining visual information, supporting memory formation and recognition of familiar visual cues or environments. The presence of theta oscillations is thought to reflect the neural mechanisms involved in integrating sensory input and storing visual memories [143–145]. A recent study found that visual familiarity-evoked theta frequency oscillations in *Fmr1 KO* mice were attenuated and frequency shifted [146]. Specifically, these oscillations were weaker in magnitude and demonstrated fewer and lower amplitude oscillation peaks compared to WT mice. They were also 0.7 Hz slower. These findings suggest deficits in encoding familiar stimuli in the primary visual cortex (V1) of *Fmr1 KO* mice [146]. Consequently, a decrease in the theta frequency may lead to the information not being transmitted efficiently between different brain areas in *Fmr1 KO* mice. This in turn may lead to the information not being encoded. It is not currently clear why the frequency of theta oscillatory activity is decreased in *Fmr1 KO* mice, but the previous research has described decreased resonance frequency of the layer 5 intrinsically bursting neurons in *Fmr1 KO* brain slices of the primary somatosensory cortex (S1) [147]. This resonance frequency shift was caused by the reduction and dysfunction of dendritic h- and BKCa channels [147]. Resonance is the optimal frequency leading to the maximal response of neurons to oscillatory current input. It is dependent on the passive membrane and channel properties of neurons [148]. Hyperpolarization-activated cyclic nucleotide-gated Channel 1 (*HCN1*), the channel mediating the h-current, is expressed extensively in apical dendrites and is critical for the nonselective cation currents triggered by hyperpolarization [149, 150]. L5 pyramidal neurons demonstrate theta resonance following optogenetic stimulation of PV^+^ interneurons in the neocortex, and this resonance depends on *HCN1* channels, too [151]. Interestingly, the resonance frequency shift in the apical dendrites of L5 pyramidal cells perfectly matches the theta frequency shift observed in *Fmr1 KO* mice during visual familiarity experiments.

Low-frequency theta oscillations have been hypothesized to represent a mechanism of inter-areal communication between V1 and higher-visual areas (HVAs), which may also include top-down modulation of V1 [145, 152, 153]*.* Visual familiarity-induced theta oscillations might represent the mechanism by which different brain areas are synchronized to allow for the processing of familiar content [145]. This oscillatory activity and multi-areal synchronization may be specific for certain features of the visual stimuli and may involve distinct information processing streams [145].

#### 3. Link between disruption in interneuron function, impaired intracortical dynamics and deficits in visual perception and learning

As the FXS field emphasizes dysfunctional inhibition as an underlying cause of many symptoms associated with FXS (and likely ASD), several studies have used animal models, particularly the *Fmr1 KO* mouse to examine the specific contribution of three major inhibitory cells: parvalbumin (PV^+^), somatostatin (SST^+^) and vasoactive Intestinal Peptide (VIP^+^). Studies in mice also show a strong influence of E-I balance on the development of cortical network activity and establishment of interneurons in an activity dependent manner [154–156]. Further, links between disruption in inhibition and perturbed neural rhythms have been shown in the context of FXS. These reasons underscore the need to examine the contribution of specific inhibitory cell types to sensory issues.

### Parvalbumin and Somatostatin neurons

PV^+^ neurons make up ~ 40% of inhibitory cells and by synapsing onto the soma of excitatory cells, provide fast and robust inhibition of pyramidal cell output. In contrast, SST^+^ cells synapse onto pyramidal cell dendrites, regulating and integrating synaptic input. Therefore, deficits in PV^+^ and/or SST^+^ function can have deleterious effects on circuit function. For instance, abnormal function of parvalbumin positive (PV^+^) GABA neurons in the cortex can result in a decrease in low-gamma oscillations and an increase in broadband gamma power [88, 91–93]. Additionally, deletion of *Fmr1* in PV^+^, but not somatostatin-positive (SST^+^) neurons, resulted in abnormal anxiety and social behaviors, and dysregulated de novo protein synthesis [94]. Analyses of functional and synaptic connectivity have demonstrated decreased excitatory drive onto inhibitory cells, including in the primary somatosensory cortex (S1) [157] and V1 of *Fmr1 KO* mice [146]. The hypoactivation of PV^+^ cells in *Fmr1 KO* mice aligns with these findings and can be rescued by the cell-specific activation of PV-cell activity using designer receptors exclusively activated by designer drugs (DREADDs), resulting in improved performance in a visual discrimination task [158]. Targeting Kv3.1 channels, which are associated with PV^+^ neurons, during specific developmental windows has been shown to enhance circuit and behavioral function [159]. In vitro studies found that hypofunctioning SST^+^ cells impair pyramidal cell spike synchrony, affecting cortical dynamics [160]. Therefore, hypoactivation of inhibitory neurons may lead to a shift in the excitatory-inhibitory (E-I) ratio and contribute to the cortical hyperexcitability observed in FXS and various forms of ASDs. However, we cannot exclude the possibility that the hypoactivity in interneurons represents a homeostatic compensatory mechanism in response to decreased excitatory drive [161].

During early development in mice, majority of PV^+^ cells that migrate to the cortex originate in the medial ganglionic eminence (MGE). In the auditory cortex, there is delayed development of PV^+^ GABA neuron density in *Fmr1 KO* mice [95]. In addition, both PV^+^ cells and their developmental precursors are hypoactive [159]. This aberrant development, disrupts the coupling between inhibitory and excitatory cells, altering the E-I balance. Interestingly, increasing the activity of immature PV^+^ cells, early in development, restores density of PV^+^ cells, but not adult neural circuit function [159]. Perineuronal nets, often observed in the context of PV^+^ cells, are extracellular matrix structures that form a net or a mesh around the soma, axon initial segments and proximal dendrites of PV^+^ cells [162, 163]. Alterations in PNNs have been shown in a number of neurodevelopmental and neurodegenerative disorders [96, 164]. There is also abnormal development of the perineuronal nets (PNN) that are associated with PV^+^ neurons in the auditory cortex. This is associated with enhanced spiking of individual neurons in the auditory cortex to sounds. Loss of *Fmr1* leads to elevated activity of matrix metalloprotease-9 (MMP-9). Genetic or pharmacological reduction of MMP-9 in *Fmr1 KO* mice restores PNN density on PV^+^ neurons and normalizes spiking activity of single neurons and EEG phenotypes [95–97]. Particularly *FMRP* re-expression only in excitatory neurons between postnatal day 14–21 improved PV cell density and their activation [102] Taken together, sensory cortical data in *Fmr1 KO* mice show abnormal expression and function of PV^+^ GABA neurons across development. These deficits may underlie the EEG circuit dynamics seen in *Fmr1 KO* mice and FXS humans. Targeted restoration of PV^+^ neuron function shows improved functional and behavioral outcomes.

#### Vasoactive intestinal peptide neurons

VIP^+^ cells, expressing calretinin (CR +), predominantly inhibit other interneurons, with strong inhibition provided to somatostatin (SST^+^) cells and weaker inhibition to parvalbumin (PV^+^) cells [165]. By inhibiting other interneurons, VIP^+^ cells mediate disinhibition of excitatory pyramidal (Pyr) cells in the sensory cortex, hippocampus, and amygdala[165]. Through this disinhibitory circuit (VIP^+^—|SST^+^—|Pyr), VIP^+^ cells, “release” some Pyr neurons from inhibition and increase the output of Pyr cells, facilitating task engagement and associative learning [166–168]. In line with this idea, there is growing evidence that complex dynamics between PV^+^ cells, SST^+^ cells and VIP^+^ cells regulate arousal, sensory processing and perception. Multiple studies in neurotypical mice, show that VIP^+^ cells are recruited during errors made in a sensory-driven task [117, 168]. Further, VIP^+^ interneuron activity increases in response to increased arousal [169], and other goal directed behaviors [170–172] and particularly in V1, they influence visual perception [173, 174]. Thus, VIP^+^ neurons are ideally suited to encoding changes in context and brain state, thus affecting learning, attention and task performance. Given the influence of VIP^+^ cells on circuit function and behavior, one study found a correlation between dysfunction in VIP^+^ cells and susceptibility to irrelevant stimuli in *Fmr1 KO* mice [117]. Atypical VIP^+^ development is shown to prevent cortical state transitions, reduce orientation selectivity of regular spiking neurons and deficits in performing a visual discrimination task [175]. Further, a mouse model of Rett Syndrome revealed, dysfunctional VIP^+^ cells, impaired cortical dynamics, reduced modulation of brain state and deficiencies in performing social tasks [176].

In V1, VIP^+^ cells are a post synaptic target of subcortical cholinergic afferents from Nucleus Basalis (NB) [169] and Anterior Cingulate Cortex (ACC). ACC provides top-down modulation from frontal cortical areas, thus impacting attention and predictive processing. Cholinergic modulation from NB influences arousal [177, 178] and attention [179]. Cholinergic activation elevates sensory cortical activity [177, 180–182] and disruption of cholinergic activity impairs task performance that requires attention [179]. Therefore, the cholinergic system is involved during changes in arousal and detecting changes in sensory environment. Hyperfunction in the cholinergic system is reported in several studies in *Fmr1* KO mice [183–185]. A hyperactive cholinergic system can contribute to learning, hypersensitivity and anxiety issues reported in *Fmr1 KO* mice [158, 185].

Thus, VIP^+^ cells could serve as “coincidence detectors”, detecting top down and bottom up inputs, and regulating learning and attention. This idea is yet to be investigated in neurotypical circuits although deficiencies in VIP function, or the communication between ACC→VIP or NB→VIP can play important roles in FXS phenotypes.

#### 4. Contribution of impaired intercortical dynamics and disruption in top down and sub-cortical modulation of V1 to deficits in visual perception and learning

Local dysfunctional intracortical dynamics can also lead to distinct cortico-cortical connectivity due to the hierarchical organization of areas in the mouse visual cortex [105, 186]. Indeed, previous work describing layer-specific alterations in visual responses to the oddball paradigm in FXS mice is also consistent with these reports, potentially providing insights into the circuit mechanisms underlying these impairments [187]. Further, long range afferents into V1, specifically modulation from top down and subcortical areas is important in perceptual learning. Top down modulation from frontal cortical areas allows visual cortical neurons to maximize their responses to behaviorally relevant stimuli and discard input from competing distractors [188–190]. While subcortical modulation improves the reliability of V1 responses thus aiding detection of sensory stimuli and making learned associations [169, 182, 191–193].

One specific frontal cortical area–anterior cingulate cortex has been implicated in tasks involving attention, detecting change in stimuli, error detection, and contributing to the emergence of stimulus selective responses [194–197]. Recent studies in mice have shown that stimulation of ACC in mice, selectively modulates visual processing [174] and viral tracing studies identified projection neurons in ACC that densely innervate V1 [198]. Studies in humans with FXS and ASD have shown disruptions in ACC activity [199], reduced AAC activity during attentive tasks [200], and reduced glutamate metabolism in ACC [201]. FXS and ASD is associated with hypersensitivity to sensory stimuli and impairments in ACC function. However, the contribution of ACC afferents on sensory processing in V1 or its influence on VIP cells in FXS remains to be investigated. Specifically, basal forebrain stimulation enhanced the magnitude and reliability of orientation specific responses in V1 [202, 203] and optogenetic activation of cholinergic input to V1 improved visual discrimination learning by reducing the variation over trials [182]. Several studies in *Fmr1 KO* mice show overactive cholinergic signaling [183–185] including deficits in learning [185] which can contribute to anxiety, repetitive behavior, and hypersensitivity.

#### 5. Dendritic dysfunction accompanies visual perception and cognitive impairments.

Beyond the changes in synaptic plasticity and functional synaptic connectivity, *Fmr1 KO* mice exhibit a range of structural synaptic abnormalities, including an increased ratio of immature synapses, unstable dendritic filopodia, higher dendritic spine turnover ratio [204, 205]. Dendritic spines are known to demonstrate extensive structural plasticity, enabling neurons to modify synaptic connectivity, adaptively remodel neural circuits, and support learning and memory processes at cellular and molecular levels [206]. Sensory experience, even in adulthood, can shape the neural wiring by forming and eliminating synapses [207]. As the dendritic spine matures under the influence of synaptic activity, it becomes shorter, and its head becomes larger, but the neck becomes shorter and wider [208, 209]. Dendritic spines in individuals with FXS often exhibit abnormal morphology, which is linked to cognitive deficits and intellectual disability. These spines are actually dendritic filopodia, which are characteristically long and thin, indicating a lack of maturity essential for effective synaptic signaling. This immature spine morphology is consistent with impaired synaptic plasticity, which can also explain difficulties in learning and memory formation [210–212].

## Somatosensory modality

As described in other modalities, sensory abnormalities are often observed in somatosensation. Moreover, in infants who will later be diagnosed with ASD, sensory deficits (especially in visual reception) are already present at six months of age, while hallmark features of ASD —social-communication deficits, and repetitive behavior —may not be evident until 12 months of age or later [2, 213]. Thus, sensory abnormalities may serve as one of the earliest risk factors of ASD, and notably, touch is the first sensory system to develop [214].

The vast majority of studies investigating the prevalence of tactile abnormalities in ASD have used parent and/or teacher reports of behaviors associated with abnormal responses to sensory stimuli on questionnaire studies. The Dunn’s Sensory Profile (SSP) questionnaire [215] is one of the most commonly used tools to evaluate sensory perception in children with ASD. Specifically, seven questions of the SSP, based on the frequency at which their child performed these behaviors, make up the tactile sensitivity subscale of the SSP. However, some research on sensory function features direct measures via experimental studies of thresholds for detecting touch on the skin on various body parts (arm, finger, hand) using different types of passive or active stimulation (flutter, vibration, constant) at varying frequencies, amplitudes and duration [216] and brain-imaging or MEG recordings in response to tactile stimulation. Behavioral measures of touch sensitivity also include the Semmes Weinstein von Frey Aesthesiometer for Touch Assessment test. In this test, the participants report sensing contact made via a filament of various thicknesses to the skin on the left or right arm [217]. Detection of touch with the thinnest filament will be associated with high tactile sensitivity.

Although no relationship has been found between autism severity and sensory symptom severity [6], both children with FXS and children with ASD have significantly more sensory symptoms and are more impaired in tactile sensitivity than typically developing children [7]. FXS males can exhibit higher tactile defensiveness (63 vs 19%), defined as an aversion to touch or tactile stimuli, than males with ID alone [218]. Additionally, children with FXS have been described as under reactive/seeking stimulation, with a tactile-specific example being characterized as a tendency to touch people and objects [7]. Similar to humans, *Fmr1 KO* mice exhibit more atypical behaviors in response to touch/tactile stimuli. In response to social touch interaction (face-to-face) versus object touch, *Fmr1 KO* mice show higher rates of avoidance running, hyperarousal, and aversive facial expressions [219]. These mice are also unable to discriminate between novel and familiar objects, where novelty is defined by texture differences, and appropriately inhibit their startle response to a 125-dB noise (pulse) when the startle noise was preceded by a light air puff (prepulse) [220]. Additionally, they exhibit a decrease in the whisker sampling duration and a reduction in the whisker touch-time in the gap-crossing whisker-dependent behavioral paradigm [221].

### Similarities in the somatosensory cortex structure and function in humans and mice

The somatosensory system in mammals contains cortical areas devoted to the processing of touch sensory information conveyed from different parts of the body (receptors located in the skin, muscle, and joints). Touch is considered one of the most basic ways to sense the external world, and humans use it to recognize objects, discriminate textures, and develop several social aspects, such as communication and social bonding. In addition to these functions, rodents may predominantly use touch information for navigation [222, 223]. The whiskers are the critical touch organ in rats and mice, whereas in humans and other primates, the fingertips are equivalent. The organization of the peripheral somatosensory pathways in mice and humans is similar. Initially, sensory information collected from peripheral receptors located in the body and the face is transduced into electrical signals at first-order sensory neurons (dorsal root ganglion cells and the trigeminal ganglion cells). These signals are then transmitted to the central nervous system via afferent axons to intermediate brain regions (in the spinal cord and brainstem), before being transferred to specialized somatosensory nuclei of the thalamus, which functions as a relay station for all sensory stimuli. Tactile information is then sent to the subcortical and cortical brain areas (such as the primary somatosensory cortex and other somatosensory processing areas) for integration and codification.

In mice, the somatosensory system is dominated by the touch input coming from the facial vibrissae (whiskers) of the whisker pad to distinct anatomical structures in the primary somatosensory cortex (named barrels) that represent single facial whiskers [224]. This topographic organization of the somatosensory cortex is shared in both species; the barrel cortex in mice and the homunculus in humans [225]. This means that neighboring sites on the skin are represented at neighboring sites in the cortex. Likewise, two adjacent whiskers on a mouse’s face are represented in adjacent cortical barrels. However, some important species differences exist. For example, in mice, the somatosensory cortex is anatomically simpler, featuring two distinct systematic representations of the contralateral body surface (primary and secondary), whereas in higher primates (including humans), it contains many more subdivisions. Despite this, the similarities between mice and humans are sufficient to enable the use of mouse models of ASD as a reasonable option to deduce the neurobiological basis of altered tactile/touch sensitivity in human patients.

### Causal neurobiological theories for altered touch behavioral abnormalities in ASD

Tactile abnormalities are complex and may not apply to all stimuli within the same modality. In general, tactile thresholds are generally lower for high-frequency stimuli than for low frequency stimuli. Thus, individuals can have lower tactile perception thresholds (be hypersensitive) to vibrotactile stimuli at 200 Hz but not at lower-frequency vibrations at 30 Hz, where they have non-significant trend towards having higher tactile perception thresholds (hyposensitivity) than the control group [216]. Therefore, it is crucial to reiterate that the two sensory response types: hyper- and -hypo-responsiveness, can be observed in the same ASD individuals depending on the situation and the sensory modalities involved. However, some studies suggest that hypo-responsiveness may be more prevalent and specific to ASD during early childhood [12].

### Proposed theories

The neurobiological basis for these behavioral responses —hyperresponsiveness, hyporesponsiveness, and mixed responsiveness—to tactile stimuli remains unclear; however, several theories have been proposed, as described below. The first five mechanisms have been previously suggested to contribute to atypical responses to auditory and/or visual stimuli and may be conserved across these sensory modalities.

Neural/circuit mechanisms that contribute to atypical sensory processing resulting in atypical touch reactivity behavior and sensitivity.

#### 1. Defective anatomical/functional organization

One theory for the cause of altered tactile sensory processing is primary anatomical abnormalities in the sensory processing and integration pathways of the somatosensory system, likely due to developmental defects. These abnormalities may lead to atypical functional organization or interactions among components. Consequently, these defects can result in secondary alterations in sensory processing, which may manifest as conditions of sensory modulation, sensory discrimination, and sensory-based motor phenotypes, ultimately leading to atypical behavioral reactivity to touch stimuli.

#### 2. Atypical top-down and bottom-up sensory processing

Humans use incoming sensory information (bottom-up processes) and inferences from prior experience and context (top-down processes) [226] to make sense of the external world. Top-down modulation is proposed to function to prevent information overload by filtering expected stimuli for less thorough processing and new stimuli for full processing. It has been proposed that perception in ASD involves impaired top-down modulation of incoming stimuli and an over-reliance on bottom-up sensory perception [227, 228]. Thus, all incoming stimuli would be processed as unexpected, resulting in enhanced sensitivity. Likewise, an over-development of bottom-up perceptual operations is expected to enhance stimulus detection and discrimination [229].

Neurons of layer (L) 2/3 of the primary somatosensory cortex encode touch and whisker movements and integrate bottom-up sensory signals originating in the periphery with information arriving from higher cortical areas [230]. In vivo electrophysiological recordings in the primary somatosensory cortex of *Fmr1 KO* mice reveal several functional abnormalities in L2/3 that could potentially impair sensory signal integration, including broadening of receptive fields in response to whisker stimulation [161, 221], abnormal encoding of tactile stimuli at different frequencies [221] and reduced whisker related activation of receptive fields [231].

#### 3. Abnormal habituation levels within the somatosensory system

Hypersensitivity may result from changes at various sensory processing stages, including peripheral receptors in the skin, sensory neurons in the intermediate brain regions (such as the spinal cord and brainstem), somatosensory nuclei of the thalamus, or neurons of the primary somatosensory cortex and other somatosensory processing areas. One theory suggests that a lack of habituation in neural pathways, which typically occurs after repeated exposure to a sensory stimulus, could explain tactile hypersensitivity [232].

Evidence supporting this theory is seen in *Fmr1 KO* mice. Quantification of in vivo two-photon imaging of GCaMP6s signals in L2/3 neurons of the primary somatosensory cortex in control and *Fmr1 KO* mice revealed that while a smaller proportion of neurons responded to whisker stimulation, a larger neuronal population was not-locked to stimulus epochs. Notably, *Fmr1 KO* neurons that were not time-locked to the stimulus exhibited an impaired ability to decrease activity in response to repetitive whisker stimulation [231]. The authors suggest that this impaired neuronal adaptation to repetitive tactile stimuli may contribute to tactile defensiveness. In juvenile, head-restrained, awake *Fmr1 KO* mice, defensiveness was characterized by an escape response, such as increased locomotion on a floating polystyrene ball treadmill, in response to repetitive whisker stimulation delivered by a flexible wire stimulator controlled by a piezoelectric actuator [231]. While in late adolescence, it manifested as increased steering away from the source of stimulation [231].

#### 4. Increased ratio of excitation to Inhibition results in high levels of noise contributing to abnormal sensory cortical maps

This theory proposes that some forms of ASD are caused by an increased ratio of excitation to inhibition in sensory, mnemonic, social, and emotional systems of the brain [233]. In the sensory cortex, this increased excitation-inhibition ratio can arise from genetic, environmental, and/or physiological processes, leading to elevated levels of neural noise (i.e. hyper-excitability). This disruption in the balance of excitation and inhibition may impair the normal formation of sensory cortical maps and predispose the cortex to epilepsy and other developmental changes that contribute to brain dysfunction observed in ASD.

#### 5. Contribition of reduced inhibition in Peripheral Sensory Neurons

GABA_A_ receptors (GABA_A_Rs) are ligand-gated chloride channels crucial for synaptic inhibition in the brain. They consist of diverse subunits that form various subtypes with different sensitivities to GABA and pharmacological properties. The β3 subunit, encoded by the *GABRB3* gene, is the primary isoform during brain development and is strongly associated with ASD [234]. In male mice with heterozygous deletion of *Gabrb3*, lower withdrawal thresholds to von Frey filaments applied to the planter surface of the hind-paw were observed compared to controls [234]. However, this phenotype was not solely explained by haploinsufficiency of *Gabrb3*, as its severity was significantly influenced by whether the *Gabrb3* gene disruption was on the paternal or maternal allele.

In humans, the link between tactile sensitivity and genetic variation in *GABRB3* is more complex. Three single nucleotide polymorphisms (SNPs) in the *GABRB3* gene (rs11636966, rs8023959, and rs2162241) were associated with lower Sensory Sensitivity Profile (SSP) scores compared to controls. Although lower, these scores remained within the normal sensitivity range, with an average SSP score of 32.36 ± 2.80 (normal range: 30–35). Despite this, behavioral touch tests using the Von Frey Aesthesiometer revealed significantly lower scores. Futhermore, the exact functional impact of these SNPs on *GABRB3* gene expression remains unknown [217].

In the *Gabrb3* ASD mouse model, peripheral mechanosensory neurons, specifically low-threshold mechanoreceptor neurons and their spinal cord connections, exhibit dysfunction due to a loss of GABA_A_ receptor-dependent presynaptic inhibition [220]. Subsequent studies explored whether enhancing GABA_A_R signaling in peripheral sensory neurons could attenuate tactile over-reactivity in various ASD models [235]. Acute treatment with a peripherally restricted GABA_A_R agonist isoguvacine (2 mg/kg, i.p.) reduced hypersensitivity in hairy skin, as measured by tactile prepulse inhibition of an acoustic startle response assay (tactile PPI) and response to air puff stimuli applied to the back of hairy skin in five genetic ASD mouse models: *Cntnap2*^lacZ/+^,*Fmr1*^*−/y*^,*Shank 3B*^±^; *Advillin*^*Cre*^*; Mecp2*^*f/y*^ and *Advillin*^*Cre*^*; Gabrb3*^*f/*+^ mutant mice. Conversely, in 16p11.2 deletion (*16p11.2*^*del/*+^) mutant mice with hairy skin hyposensitivity, isoguvacine enhanced these deficits. Thus, GABA_A_R agonists may be a viable treatment option for hypersensitivity to touch.

#### 6. Defective Emotional/Social Stimulus Processing

Rutter’s theory, proposed in 1983, suggests that sensory stimulus processing may be normal except when the stimulus is linked to social or emotional content. In these cases, the emotional response to the sensory input may lead to alterations in the processing or filtering of the sensory signal, resulting in abnormal behavioral responses to touch.

#### 7. Altered Temporal Binding of Sensory Stimuli

People with autism often struggle to process information in context, having difficulty extracting the global form and meaning, or the “big picture,” from numerous details—a phenomenon known as “weak central coherence” [236]. This means they excel at analyzing and processing specific details or individual stimuli but may struggle with integrating all input to derive the overall context. Brock et al. [237] proposed that weak central coherence arises from a deficiency in binding and integrating associated stimuli, due to defects in the temporal binding of activity within or between local neural networks. The temporal binding hypothesis suggests that sensory stimuli occurring close in time are more likely to be integrated and perceived as a whole [238]. Therefore, an extended “temporal binding window” in individuals with ASD might result in a blurred, unpredictable representation of the sensory environment, with unrelated stimuli being processed together.

#### 8. Hyperfunctioning of Neural Circuits

This theory, proposed by Henry and Kamila Markram [239, 240], suggests that behavioral symptoms such as hyper-perception, extreme sensitivity to sensory stimuli, and up-regulation in primary sensory perception in ASD result from hyper-functionality (i.e., hyper-reactivity, hyper-plasticity, hyper-connectivity, and generally upregulated activity) in local neural circuits.

## Polypharmacological therapeutic strategy for FXS

Theoretical and empirical studies described above suggest that atypical behavioral responses to touch, lights and sounds in ASD are often associated with neuronal hyperexcitability in circuits, leading to hypersensitivity to perceptual stimuli. In line with this, multiple interneuron populations have been tested as potential targets for therapy. GABA_A_R agonists are proposed as potential treatments for tactile hypersensitivity [235]. Restoring PV^+^ function in *Fmr1 KO* mice improves the tuning of visual receptive fields and rescues perceptual learning [158]. However, enhancing activity of precursors of PV^+^ cells in *Fmr1 KO* mice restored PV^+^ cell density to neurotypical levels but failed to improve circuit function [159]. While these studies provide insights into potential cell targets for rescuing sensory symptoms, they also highlight the limitations of GABA_A_R agonists as potential therapies and the complexities of FXS. Indeed, in FXS, multiple biological pathways are dysregulated, including those involving metabotropic glutamate receptors, gamma-aminobutyric acid (GABA), and potassium channels [241]. Notably, reductions in inhibition or increased mGluR5 activity observed in *Fmr1 KO* mice may stem from primary deficits in potassium channel function [242, 243]. Thus, by modulating upstream mechanisms, drugs targeting potassium channels may act as polypharmacological agents, simultaneously influencing several pathways implicated in sensory atypicalities.

Potassium channels play a crucial role in regulating neuronal excitability, including homeostatic regulation, intrinsic membrane properties, and action potential characteristics such as spike threshold, width, peak, and repolarization. Several potassium channels are directly regulated by *FMRP* at the mRNA and/or protein level, including the Na^+^-activated K^+^ channel (K_Na_) Slack-B, the calcium-activated potassium (BK) channel [244, 245], the voltage-gated K^+^ channels K_v_3.1b, K_v_1.2 [243], K_v_4.1[246] and K_v_4.2 [247]. Consequently, decreased expression and function of several potassium channels have been reported in neurons derived from FXS patient induced pluripotent stem cells [248] and *Fmr1 KO* mice [249–251]. In these cases, in vivo administration of potassium channel agonists/activators such as GIRK1/2 channel agonist ML297 [252], the BK_Ca_ channel opener BMS-204352 [253, 254], the BK channel activator VSN16R [255], the allosteric K_v_1.2 agonist, docosahexaenoic acid [243] and the Kv3.1-positive allosteric modulator, AG00563 (1-(4-methylbenzene-1-sulfonyl)-N-[(1,3-oxazol-2-yl)methyl]−1H-pyrrole-3-carboxamide) [159], have efficiently reversed several ASD-related phenotypes in *FMR1 KO* mice, including neuronal hyperexcitability and tactile and auditory sensory behavioral hypersensitivity.

Interestingly, potassium channel activity may exhibit a notable role in contributing to auditory hypersensitivity. In *Fmr1 KO* mice, this hypersensitivity is linked to enhanced activity in the auditory brainstem nuclei [256]. Current-clamp recordings of neurons in the medial nucleus of the trapezoid body revealed repetitive firing rather than a single AP, in *Fmr1* KO mice in response to sustained depolarization. Voltage-clamp recordings in *Fmr1* KO mice showed that K_v_ currents that activate at positive potentials to facilitate high-frequency firing were elevated, while those that activate near the resting potential and inhibit repetitive firing were reduced [256]. Treatment of brain slices with 10 μM AUT2 [(Autifony Therapeutics, ((4-({5-[(4R)−4-ethyl-2,5-dioxo-1-imidazolidinyl]−2-pyridinyl}oxy)−2-(1-methylethyl) benzonitrile], a modulator of K_v_3.1 and K_v_3.2 channels, reduced high-threshold K_v_ currents and increased low-threshold K_v_ currents in neurons by shifting activation to more negative potentials. Behaviorally, AUT2 treatments (30 mg/kg, i.p., administered 20 min before testing to juvenile *Fmr1* KO mice at postnatal day 15) were effective in normalizing the auditory brainstem response to varying sound stimuli intensities [256]. These results suggest that normalizing spiking activity through enhanced K_v_ channel activity could be a promising approach for treating sensory hypersensitivity in FXS patients.

## EEG measurements as a biomarker for abnormal speech and language processing

Temporal processing is crucial for recognizing and producing speech. EEG data from the *Fmr1 KO* rodent and FXS humans shows similar phenotypes in terms of reduced temporal processing fidelity [51, 52, 80, 257]. Specifically Ethridge et al. [52], showed that elevated gamma resting state power is correlated with reduced spectrotemporal processing for dynamic auditory stimuli in FXS. Therefore, the link between spectrotemporal processing and speech [74], suggests that abnormal gamma band power is related to abnormal speech processing. However, Wilkinson and Nelson [258] showed that the elevated high frequency power is correlated with improved language function in a cohort of children with FXS (mean age ~ 4 years). There was no association between gamma power and sensory difficulties or adaptive behaviors. Thus, while the elevated gamma power findings remain consistent across studies, the clinical implication is unclear. Taken together, the repeatability of findings around elevated gamma band resting EEG power across subject age and cohorts indicates that this phenotype may serve as a useful biomarker in FXS. Recent studies suggest that the elevated broadband gamma power reflects cortical activation and elevated E:I excitatory-inhibitory (E-I) balance in the cortex [259], particularly through abnormal activation of parvalbumin positive GABAergic neurons [88]. Indeed, dysfunctional E-I balance is a feature that is observed across modalities. Future studies should examine correlations between *FMRP* levels, baseline gamma power, and intellectual deficits as a potential method to stratify patients for clinical trials.

## Conclusion

Sensory impairments, impulsivity, and persistent inattention are among the most consistent clinical features of FXS, all of which impede daily functioning and create barriers to learning. We begin this review by emphasizing that sensory issues vary from over-responsiveness to hypo-responsiveness, and that sensory abnormalities in the somatosensory domain are manifested differently across stimuli. Thus, while the consensus in the FXS field is that dysfunctional E-I balance underlies sensory deficits across modalities, the link between impaired inhibition and sensory phenotypes remains blurry. In light of the evidence and state of FXS research, we identify the following future research needs: 1) Delineating the effect of different sub-types of inhibitory cells to impaired circuit function and both hypo- and hyper-responsiveness, 2) Focusing basic and translational research efforts in animal models of FXS can help identify dysfunctional neural mechanisms, contributing to deficits. However, to improve the translational impact of research from mouse models, continued studies using both human subjects and animal models will be essential for developing effective interventions that enhance the quality of life and learning potential for individuals with FXS and ASDs [260]. A significant step in this direction are recent studies that adapted the EEG technique in mice and show remarkably similar EEG phenotypes in *Fmr1 KO* mouse and humans with FXS [64], and a compelling alignment of sensory phenotypes in both humans with FXS and *Fmr1* KO mice using an analogous visual discrimination tasks [117, 158]. Such robust, repeatable, and scalable phenotypes can serve as translation relevant biomarkers to develop pre-clinical drug development and in human clinical trials. These responses may serve as outcome measures and/or stratification strategies. However, as we mention in the previous section, an important caveat is that the clinical relevance of such measures remains mostly understudied and should be a focus of future studies. 3) Impaired sensory perception and learning in FXS and ASD reflect complex anomalies affecting sensory processing circuits. These challenges underscore the need for tailored educational and therapeutic strategies that address the specific sensory and cognitive needs of individuals affected by these disorders (Fig.1). Currently, there is a scarcity of studies focused on the inter-areal interaction and sensory binding in ASD [261]. Pinpointing the exact neural pathways and molecular targets that could be manipulated to improve sensory processing and learning outcomes will be invaluable in designing therapies that target specific phenotypes.

## Data Availability

No datasets were generated or analysed during the current study.
